# Meeting Report: The Role of Environmental Lighting and Circadian Disruption in Cancer and Other Diseases

**DOI:** 10.1289/ehp.10200

**Published:** 2007-06-14

**Authors:** Richard G. Stevens, David E. Blask, George C. Brainard, Johnni Hansen, Steven W. Lockley, Ignacio Provencio, Mark S. Rea, Leslie Reinlib

**Affiliations:** 1 University of Connecticut Health Center, Farmington, Connecticut, USA; 2 Bassett Research Institute, Cooperstown, New York, USA; 3 Jefferson Medical College, Thomas Jefferson University, Philadelphia, Pennsylvania, USA; 4 Danish Cancer Society, Copenhagen, Denmark; 5 Harvard Medical School, Boston, Massachusetts, USA; 6 Department of Biology, University of Virginia, Charlottesville, Virginia, USA; 7 Lighting Research Center, Rensselaer Polytechnic Institute, Troy, New York, USA; 8 Division of Extramural Research and Training, National Institute of Environmental Health Sciences, National Institutes of Health, Department of Health and Human Services, Research Triangle Park, North Carolina, USA

**Keywords:** breast cancer, circadian rhythms, clock genes, lighting, melatonin, phototransduction, pineal gland

## Abstract

Light, including artificial light, has a range of effects on human physiology and behavior and can therefore alter human physiology when inappropriately timed. One example of potential light-induced disruption is the effect of light on circadian organization, including the production of several hormone rhythms. Changes in light–dark exposure (e.g., by nonday occupation or transmeridian travel) shift the timing of the circadian system such that internal rhythms can become desynchronized from both the external environment and internally with each other, impairing our ability to sleep and wake at the appropriate times and compromising physiologic and metabolic processes. Light can also have direct acute effects on neuroendocrine systems, for example, in suppressing melatonin synthesis or elevating cortisol production that may have untoward long-term consequences. For these reasons, the National Institute of Environmental Health Sciences convened a workshop of a diverse group of scientists to consider how best to conduct research on possible connections between lighting and health. According to the participants in the workshop, there are three broad areas of research effort that need to be addressed. First are the basic biophysical and molecular genetic mechanisms for phototransduction for circadian, neuroendocrine, and neurobehavioral regulation. Second are the possible physiologic consequences of disrupting these circadian regulatory processes such as on hormone production, particularly melatonin, and normal and neoplastic tissue growth dynamics. Third are effects of light-induced physiologic disruption on disease occurrence and prognosis, and how prevention and treatment could be improved by application of this knowledge.

Humans have evolved over millions of years and adapted to a solar day of approximately 12 hr of light and 12 hr of dark, latitude and season permitting. Our ability to artificially light the night began about 250,000 years ago when we discovered how to use fire. Candles were introduced about 5,000 years ago, and gas street lighting was possible beginning in the mid-1700s. However, only in the last 120 years has environmental illumination begun to change on a pervasive scale for the masses of people through the introduction of electric lighting. One of the defining features of the built environment in the modern world is this artificial lighting. Electricity has made it possible to light the inside of large buildings and light the night for work, recreation, and security. The benefits of this lighting are obvious and enormous. It has become apparent, however, that although of obvious benefit, it may not be completely innocuous. Light, including artificial light, can be potent in regulating human physiology and behavior and can therefore alter human physiology when inappropriately timed. One example of potential light-induced disruption is the effect of light on circadian organization, including the production of several hormone rhythms. Changes in light–dark exposure shift the timing of the circadian system such that internal rhythms can become desynchronized from both the external environment and internally with each other, impairing our ability to sleep and wake at the appropriate times and compromising metabolic processes. Light can also have direct acute effects on neuroendocrine systems, for example, in suppressing melatonin synthesis or elevating cortisol production that may have untoward long-term consequences.

There is limited but thus far generally consistent evidence in support of the hypothesis that altered lighting can play a role in breast cancer causation (Blask et al. 2005a; Hansen 2001; Stevens 1987, 2006), and there is growing interest in a lighting and/or sleep connection to other conditions such as prostate cancer (Kubo et al. 2006; Stevens et al. 1992), obesity (Spiegel et al. 2004), diabetes (Spiegel et al. 2005), and depression (Srinivasan et al. 2006). It may not be entirely coincidental that the dramatic increases in risk of breast and prostate cancers, and of obesity and early onset diabetes have mirrored the dramatic changes in the amount and pattern of artificial light generated during the night and day in modern societies over recent decades. The science underlying these hypotheses has a solid base and is currently moving forward rapidly.

For these reasons, the National Institute of Environmental Health Sciences (NIEHS) on 14–15 September 2006 convened a workshop of a diverse group of scientists to consider how best to conduct research on possible connections between lighting and health. The potential for circadian disruption, particularly light exposures at night, to contribute to the etiology of cancer or other diseases has become an urgent question in part because a large and increasing segment of the population of industrialized nations is working nonday shifts (Rajaratnam and Arendt 2001). It is also important because the population in general has been reducing its exposure to dark over recent decades as reflected in a decrease in the average duration of sleep (National Sleep Foundation 2005).

## Definitions

“Diurnal rhythms” denote recurring daily 24-hr rhythms in physiology, behavior, or metabolism under entrainment conditions but not necessarily under constant, non-entrainment conditions. “Entrainment” is the synchronization of a rhythm by a repetitive signal (e.g., the light–dark cycle). “Circadian rhythms” (e.g., melatonin production) denote those endogenous, near 24-hr physiologic, metabolic, or behavioral rhythms that persist under constant nonentrainment conditions (e.g., constant darkness) and are presumably driven by the molecular clockwork mechanism in the suprachiasmatic nucleus (SCN) and peripheral tissues (Dunlap et al. 2004).

## Background

### Circadian rhythms—master and peripheral clocks

The hypothalamic SCN is the master circadian pacemaker in mammals, driving circadian rhythms in behavior, physiology, endocrinology, and metabolism (Ko and Takahashi 2006; Moore and Leak 2001). The core genetic apparatus of the clock mechanism is being rapidly revealed and thus far appears to be based on a small number of genes (Fu and Lee 2003; Hastings et al. 2003). Lesions of the SCN abolish these rhythms. Such lesion studies cannot discount the possibility that the SCN is merely serving as a relay point to or from a “true” pacemaker. However, transplantation of SCN donor tissue derived from circadian period mutants into SCN-lesioned wild-type hosts resulted in a transfer of period from donor to host, thereby proving the SCN to be a true master pacemaker (Ralph et al. 1990).

**Figure 1:**
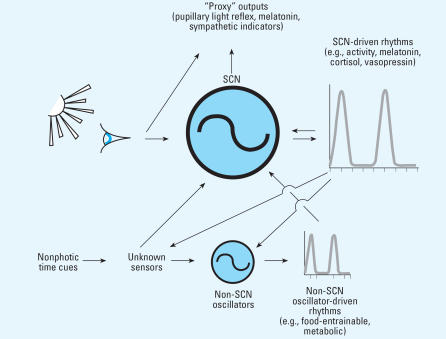
The suprachiasmatic nuclei (SCN) and other circadian oscillators drive a broad range of circadian rhythms. These rhythms, in turn, can feedback on the oscillators. In addition to this feedback regulation, the oscillators are entrained by photic and nonphotic environmental time cues.

In the early 1990s, peripheral circadian oscillators were not thought to be prevalent, existing only in the eyes of frogs and mammals (Cahill et al. 1991; Tosini and Menaker 1996). In the late 1990s, however, the use of bioluminescent reporter proteins expressed under the control of clock gene promoters revealed that circadian oscillators resided in many peripheral tissues of vertebrates and invertebrates (Balsalobre et al. 1998; Plautz et al. 1997). This concept is depicted in Figure 1. These oscillators could be entrained and phase shifted by biologically relevant stimuli such as light. Interestingly, treatments resulting in large abrupt phase shifts caused peripheral tissues to become transiently desychronized as a result of differential rates of phase shifting to the stimulus (Yamazaki et al. 2000).

Recently, the effect of chronic jet lag on aged mice was shown to have dramatic consequences on mortality (Davidson et al. 2006). Only 47% of aged mice forced to phase advance 6 hr once per week survived the experiment compared with an 83% survival rate in an unshifted age-matched control group. Aged mice subjected to weekly 6-hr phase delays showed an intermediate survival (68%). These profound effects of phase shifting were not observed in younger mice. The mechanism by which chronic jet lag hastens death in aged mice remains to be determined. The authors of the study speculate that the internal desynchrony of oscillators likely to result from a rotating light schedule may have dire consequences that may be exacerbated by age (Davidson et al. 2006). If this reasonable hypothesis is proven true, then the effects of exposure to uncontolled environmental lighting may have a detrimental impact on health, where the ultimate outcome may be much more serious than previously believed.

**Figure 2:**
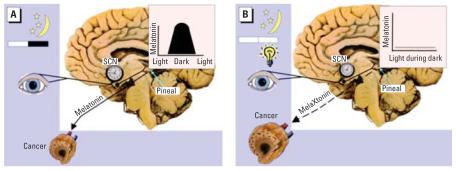
A schematic representation of the cancer response to the effects of an intact nocturnal, circadian melatonin signal (*A*) under 12-hr:12-hr light/dark conditions and (*B*) under conditions in which the melatonin signal is disrupted by ocular exposure to bright polychromatic light at night.

Experimental simulation of jet lag in mice has also been shown to increase growth of Glasgow osteosarcoma (Filipski et al. 2004). Disruption of circadian physiology by mutation or ablation of specific clock genes has also been tested for effects on cancer growth. Mutation in the Period 2 (*Per2*) gene was shown to increase susceptibility to radiation-induced lymphoma (Fu et al. 2002). Gauger and Sancar (2005), however, showed that abolishment of circadian rhythmicity alone was not sufficient to affect tumor growth by finding no increase in lymphoma after radiation exposure to cryptochrome knockout mice. In addition, exposure of rats bearing human breast cancer xenografts (or murine tumors) to light in night has been shown to stimulate tumor growth, metabolism, and signal transduction activity (Blask et al. 2005a, 2005b) (Figure 2).

### Phototransduction for the circadian system

Empiric evidence from the past three decades has confirmed that, relatively separate from vision and visual reflexes, light incident on the retina can be a potent biological, behavioral, and therapeutic stimulus in humans (Commission Internationale de L’Eclairage 2007). Recently, there has been a paradigm shift in the understanding of photoreception for circadian, neuroendocrine, and neurobehavioral responses in humans. In 2001, two action spectra for melatonin suppression in humans showed that monochromatic blue light was most potent for melatonin suppression in humans (Brainard et al. 2001; Thapan et al. 2001). Altogether, the elucidation of eight separate action spectra in rodents, monkeys, and humans showed that the short wavelength blue portion of the visible spectrum between 459–484 nm is the most potent wavelength region for a range of biological and behavioral responses in these species [for review, see Brainard and Hanifin (2005)]. Other studies have confirmed that shorter wavelength monochromatic light is more potent than equal photon densities of longer wavelength light for evoking circadian phase shifts, suppressing melatonin, and enhancing alertness in humans (Cajochen et al. 2005; Lockley et al. 2003, 2006; Revell et al. 2006). Together, the full analytic action spectra along with the selected wavelength testing indicate that a novel photoreceptor system, distinct from the visual rods and cones, is primarily involved in circadian, neuroendocrine, and neurobehavioral responses mediated by the eyes of humans and other mammals.

Studies employing both animal and human models are rapidly clarifying the neuroanatomy and neurophysiology of this photosensory system. A recently discovered photopigment, melanopsin, has been localized in the retinas of both rodents and humans (Provencio et al. 1998, 2000). More specifically, melanopsin is found in a subtype of intrinsically photoreceptive retinal ganglion cells (ipRGCs) (Berson et al. 2002; Hattar et al. 2002). These light-sensitive retinal ganglion cells extend an expansive arbor of dendrites that seems to form a “photoreceptive net” and project to the SCN via the retinohypothalamic tract for circadian phototransduction (Gooley et al. 2001; Provencio et al. 2002). Although the light detection for circadian, neuroendocrine, and neurobehavioral regulation seems to be mediated principally by ipRGCs, studies with melanopsin knockout mice have shown that the classic rod and cone visual photoreceptors nevertheless appear to have some role in modulating these responses (Hattar et al. 2002; Panda et al. 2003).

## Workshop

The NIEHS sponsored a workshop on 14 and 15 September 2006 charged with producing “a prioritized set of research recommendations that will guide future NIH [National Institutes of Health] funding agendas as well as a publication that will assist the research community in their efforts.” Participants at the workshop represented a diverse array of scientific expertise from molecular genetics of the clock mechanism in cyanobacteria to molecular biology of phototransduction in the mammalian retina, the effects of light on human physiology, circadian rhythm sleep disorders, physiologic effects of sleep on hunger, experimental carcinogenesis, epidemiology of disease occurrence, and application of circadian principles to treatment of cancer patients. Although some participants had worked together in the past, many were new to the topics of health research and architectural lighting. The meeting was highly unusual in the breadth of its cross-discipline content. Yet given the complexity of the lighting, circadian rhythms, and health subject matters, and the importance of the diseases that might be involved, this broad expertise was required for engendering productive interaction among participants to ensure that the mandate of the workshop was met.

The most studied environmental exposure that can disrupt circadian rhythms is light. However, other factors are emerging that have this potential and include the timing of meals (Filipski et al. 2005; Mendoza 2007), stress (Funk and Amir 1999), and alcohol (Allen et al. 2005). It is only recently being investigated how these can interact with light exposure. To the extent that early-life exposures, even *in utero* (Hilakivi-Clarke and de Assis 2006), can affect lifetime risk of cancer, maternal exposure to altered lighting, and these other potential circadian disruptors need to be investigated.

## Recommendations

The short-term goal of the workshop was to lay the foundation for the development of a better understanding of the extent to which changes in the lighted environment that come with industrialization contribute to the etiology and affect the risk of human disorders such as breast cancer, other cancers, and other chronic diseases. Another workshop objective was to foster an understanding of the role that light plays in conjunction with other environmental and endogenous factors (e.g., fatty acids) that may affect cancer development and growth. Of primary concern are the altered light exposures during the night and day as they impact circadian and diurnal physiologic rhythms. It is also a priority to understand the biological mechanisms connecting disruption of circadian and diurnal processes with disease etiology. Importantly, characteristics of the individual that might modify the effect of environmental lighting on health need to be elucidated. The long-term goal is, of course, to use the newly acquired knowledge of the mechanisms and consequences of circadian disruption from altered lighting to develop interventional strategies and technologies for reducing health risks from, for example, non-day shift work in which a large and increasing segment of the population is employed.

There are three broad areas of research effort that need to be addressed. First are the basic biophysical and molecular genetic questions of how the retina transduces the photic input to a neuronal signal to the circadian system and other brain neural substrates that mediate non-image-forming effects of light, and once received, how these signals are transduced to other cells and tissues of the body. It has become clear that photic sensing for the circadian, neuroendocrine, and neurobehavioral effects of light is not by vision per se but via a novel photoreceptor system located in the ganglion cell layer of the retina. Fundamentally, the basic biophysics of nonvisual phototransduction needs to be understood, that is, its absolute, spectral, spatial, and temporal response characteristics. Among the interesting questions identified: Do the absolute and spectral sensitivities change throughout the solar day? Does a single spectral sensitivity characterize the photic input to every light-responsive, nonvisual system of the brain? How does prior light exposure affect light sensitivity (Hebert et al. 2002)? And, significantly, how can basic knowledge be used to develop lighting regimens or sources that mitigate the adverse consequences of altered light exposure, for example to reduce circadian misalignment in shift workers?

Second are questions of physiologic consequences of disrupting the normal functioning and temporal synchronization of the circadian and other neurobehavioral systems: How are hormone production and release affected both acutely and chronically? How are the timing and synchronization of metabolic processes affected? How does altering clock gene function affect cell cycle checkpoint genes and genes of apoptosis in susceptible tissue such as breast and prostate? These questions can be addressed in laboratory studies in experimental models and in clinical trials with human subjects with end points such as circulating hormone level alterations over the course of a 24-hr period.

**Figure 3:**
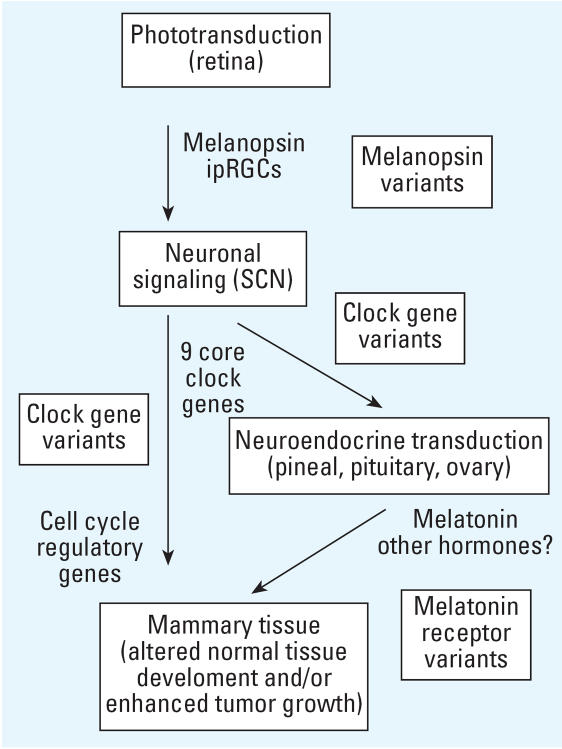
Model for mechanisms for a light-induced effect on breast cancer [adapted from Stevens (2005)]. Possible targets for research on genetic polymorphisms that might affect the process are indicated.

Figure 3 shows an integrative model for a light-induced effect on breast cancer etiology [adapted from Stevens (2005)]. Specific genetic polymorphisms may act at several points along this proposed causal pathway. There has been one study published based on this scheme that reported increased risk of breast cancer in young women with a polymorphism in one of the clock genes, *Per3* (Zhu et al. 2005).

Third are questions of disease occurrence, prevention, and treatment, which must be addressed with epidemiology and clinical trials. For example, can risks for specific diseases such as breast cancer be quantified in terms of light exposure, and can this information be used to mitigate exposure and lower risk?

Twenty-five attendees of the NIEHS workshop identified five core areas of priority research effort. These are all closely intertwined with one another and directed toward the larger goal of understanding how light and dark influence human health in the modern world. Each core area requires expertise from different but overlapping scientific disciplines, and cross-disciplinary communication among researchers in the five areas is paramount.

## Priority Research Foci

In the next section we discuss the five priority core research topics identified by the workshop participants that should be addressed in order for progress to advance on the important environmental issue of lighting and health. There are dramatic changes in the health profile of populations as societies industrialize and begin increasing their use of electric lighting. At the same time, risks of certain cancers rise (e.g., breast and prostate), as does the prevalence of obesity and early-onset diabetes. Reasons for these increases are only partly understood, and all of them are likely to be multifactorial. Altered environmental lighting during the day and night may play a large role in some diseases, an ancillary role in others, and perhaps no role in others. A crucial aspect of research in this area is accurate quantification of the light exposure (Bierman et al. 2005; Bullough et al. 2006; Figueiro et al. 2006).

To effectively address the theory that light-at-night increases cancer risk or risk of other diseases, a wide array of study designs is required (Roenneberg and Lucas 2002). Both epidemiologic and experimental approaches need to be conducted on each of the many aspects of the idea, but alone both the epidemiology (Poole 2002) and the experimental models (Anisimov 2005) have limitations in causal assessment for human risk. In particular, no one set of epidemiologic approaches is adequate nor could they prove a cause and effect exists. For example, a sufficient number of high-quality epidemiologic findings of increased risk of breast cancer in shift-working women may lead to the conclusion that shift workers are indeed at higher risk, yet they cannot prove that light-at-night (LAN), or more generally, circadian disruption, is the reason (Stevens 2006). First, nonday shift workers are different in many ways from day workers, thus presenting the potential for confounding; and second, even if the cause is shift work itself, there are other aspects of working nonday shifts apart from circadian disruption. For a conclusion of causality, the LAN theory must be tested in many different settings with very different specific predictions, and consistency seen across these studies (Hill 1965).

We do not believe, however, that research on potential interventions should be postponed until the risk has been unequivocally quantified; the two research directions should both proceed. In addition, for useful intervention strategies to be identified there needs to be a detailed understanding of the biological mechanism provided from experimental studies in animal models, cellular systems, and in humans.

## Specific Research Directions and Needs

As with any research agenda, a complex biology must be reduced to specifics. This is a perilous process. As Einstein was reputed to have said “Make things as simple as possible, but not simpler.” Circadian biology is not simple nor are the mechanisms by which lighting could disrupt the circadian rhythms or other elements of human physiology. Yet there is a simple truth that certain diseases become much more common as industrialization occurs in societies. And so, we have accepted the challenge to make concrete research suggestions while at the same time risking over simplification and/or unintentionally ignoring important aspects of the problem.

### Biophysical and physiologic mechanisms of ocular phototransduction for the circadian, neuroendocrine, and neurobehavioral effects of light

- What are the basic mechanisms of circadian, neuroendocrine, and neurobehavioral phototransduction in humans and in animal models?
- What are the parameters of the photic input that affect these processes?
- What are the patterns of light and dark exposures that people experience in everyday life and how can they be parametrically studied in humans and with experimental models?

### Molecular and physiologic mechanisms underlying circadian and diurnal rhythmicity—hierarchical and nonheirarchical mechanisms for synchronicity of master and peripheral clocks

- How does the master circadian clock in the SCN communicate with the peripheral oscillators in response to ocular light exposure?
- Given that there are multiple circadian clocks in the body, how does disruption of their timing by light affect disease?
- How does the relationship between internal clocks and environmental light affect disease?

### Testing specific disease-association hypotheses for disruption of circadian and diurnal rhythms

- At present, diseases of primary interest are breast cancer, prostate cancer, obesity, diabetes, and depression. Is their onset, maintenance, or recurrence a result of disrupted circadian or diurnal rhythms?
- Given the state of the science, what epidemiologic studies are needed to define the light exposure/disease relationships?
- What practical/feasible markers of disruption (that can be used in large numbers of humans) need to be validated in the clinic/ laboratory for use in epidemiologic studies?
- Given the emerging evidence that “clock” genes control the expression of a significant amount of the genome, which polymorphisms in them can be evaluated for disease associations? A resequencing effort for their population variability should be undertaken.
- What gene–environment interactions between clock polymorphisms and exposures (such as shift work) are important in disease susceptibility?
- Does accelerated tumor growth due to light exposure at night have the same increased wavelength sensitivity as the other circadian, neuroendocrine, and neurobehavioral responses?
- What intensities of polychromatic light exposures contribute to tumorigenesis and tumor growth?
- What is the balance of ipRGC, rod, and cone cell mediation of the effect of light on tumorigenesis and tumor growth?

### Role of endogenous melatonin in mediating light-induced–associated disease and development of exogenous melatonin therapy to prevent or alleviate light-induced disease

Although the human circadian system encompasses a vast array of physiologic responses, the hormone melatonin deserves particular attention for a possible connection to cancer for two reasons (Blask et al. 2005a, 2005b; Figure 2). First, melatonin is the biochemical signal for darkness, and its timing, duration, and amplitude respond to seasonal changes in photoperiod, specifically night-length. It is therefore the primary vehicle by which light information is translated into a biological signal and, as such, is a primary candidate for assessing the effect of light on individuals. A great deal of information is available on melatonin parameters under a variety of lighting conditions. Its strong signal and availability either as melatonin or its metabolites in blood, saliva, and urine make it a good marker for a range of studies. Second, melatonin has well-established oncostatic properties in experimental *in vitro* and *in vivo* models that can be studied in their own right as a potential mediator in disease development or through administration of synthetic melatonin as an adjuvant therapy for light- or melatonin-associated diseases. In addition to melatonin’s direct oncostatic properties, the endogenous melatonin signal may thwart cancer development and growth via indirect mechanism’s involving its ability to enhance immune activity and mitigate against stress-induced immune suppression (Maestroni 1993; Sephton and Spiegel 2003).

- What defining aspects of light (intensity, spectrum, spatial pattern, duration, and timing) exposure at night incur the highest and the least cancer risk in humans via circadian/melatonin disruption?
- What are the relative short- and long-term contributions of light in night-induced suppression of the nocturnal circadian melatonin signal versus its circadian phase disruption to the cancer initiation, growth, progression, and invasion/metastasis?
- What is the “balance” between circadian melatonin signal strength (i.e., phase, amplitude, duration), melatonin receptor expression/sensitivity, and dietary tumor stimulatory/inhibitory (i.e., linoleic acid/ omega-3 fatty acids) factors in cancer-susceptible target tissues that determines whether these tissues will be at more or less risk for developing cancer in response to circadian disruption of melatonin by light at night?
- What are the interactions between the nocturnal circadian melatonin signal and its disruption by light at night, melatonin receptors, and cancer clock genes that may be involved in regulating oncogenesis and/or tumor suppressor genes?
- What does light in night-induced immune suppression contribute to cancer development and/or growth via melatonin suppression and/or through nonmelatonin, stress-related mechanisms?
- Because the timing and amount of melatonin are regulated by light, and that it is both a chronobiotic and anticancer agent, how does disruption of melatonin by light, and use of pharmacologic melatonin supplementation as a chronobiotic and anticancer agent impact the risk and progression of cancer?
- What improvements in melatonin measurements can be made so that melatonin will be a more useful biomarker in epidemiologic studies?
- What are the optimal standards for measuring melatonin?

### Development of interventions and treatments designed to reduce the impact of environmental lighting on disease

- Can disruption of circadian, neuroendocrine, and neurobehavioral processes be ameliorated to treat or prevent disease?
- How can exposure to light and darkness be optimized to reduce the risk of cancer and other diseases, for example in shift workers?
- What are the other candidate interventions (e.g., melatonin supplementation, dietary modifications, therapeutic drugs)?
- What are the relative contributions of and interactions between the circadian and homeostatic processes of sleep/wake regulation, and light at night-induced circadian disruption in disorders of energy metabolism as related to cancer, obesity and type II diabetes? What circadian-based interventions can be implemented to prevent or treat such disorders?

## Summary

One of the defining characteristics of life in the modern world is the altered patterns of light and dark in the built environment made possible by use of electric power. A rapidly growing and very exciting body of basic science is uncovering the mechanisms for phototransduction in the retina for environmental control of circadian and other neurobehavioral responses and the makeup and functioning of the clock physiology that exert genetic control of the endogenous rhythms. It is beginning to be realized by the larger scientific community that maintenance of these circadian rhythms is important to health and well-being. Our challenge for the future is to integrate the basic science with studies in experimental animals and clinical and epidemiologic research to advance our understanding of the impact of circadian disruption from lighting, and what then can be done to minimize or eliminate the adverse consequences for human health.

## Appendix. Meeting Participants

**Table:**

David M. Berson
Brown University
David E. Blask
Bassett Research Institute
Christopher A. Bradfield
University of Wisconsin
George C. Brainard
Thomas Jefferson University
Scott Davis
Fred Hutchinson Cancer Research Center
Margarita L. Dubocovich
Northwestern University
Marie Dumont
University of Montreal
Charmane I. Eastman
Rush University Medical Center
Susan S. Golden
Texas A&M University
Johnni Hansen
Danish Cancer Society
William J. Hrushesky
WJB Dorn VA Medical Center
Cheng Chi Lee
University of Texas-Houston Medical School
Steven W. Lockley
Brigham and Women’s Hospital
Elizabeth Maull
NIEHS
Chisato Nagata
Gifu University Graduate School of Medicine
Plamen Penev
The University of Chicago
Ignacio Provencio
University of Virginia
Mark S. Rea
Lighting Research Center, Rensselaer Polytechnic Institute
Leslie Reinlib
NIEHS
Leo Smith
International Dark-Sky Association
Richard G. Stevens
University of Connecticut Health Center
Patricia A. Wood
WJB Dorn VA Medical Center
Yong Zhu
Yale University
